# A life course approach to injury prevention: a "lens and telescope" conceptual model

**DOI:** 10.1186/1471-2458-11-695

**Published:** 2011-09-08

**Authors:** Jamie Hosking, Shanthi Ameratunga, Susan Morton, Danilo Blank

**Affiliations:** 1 Section of Epidemiology and Biostatistics, School of Population Health, University of Auckland, Auckland Mail Centre, Auckland 1142, New Zealand; 2 Centre for Longitudinal Research, School of Population Health, University of Auckland, Auckland Mail Centre, Auckland 1142, New Zealand; 3 Departamento de Pediatria, Faculdade de Medicina, Universidade Federal do Rio Grande do Sul (UFRGS), Porto Alegre, RS, Brazil

## Abstract

**Background:**

Although life course epidemiology is increasingly employed to conceptualize the determinants of health, the implications of this approach for strategies to reduce the burden of injuries have received little recognition to date.

**Methods:**

The authors reviewed core injury concepts and the principles of the life course approach. Based on this understanding, a conceptual model was developed, to provide a holistic view of the mechanisms that underlie the accumulation of injury risk and their consequences over the life course.

**Results:**

A "lens and telescope" model is proposed that particularly draws on (a) the extended temporal dimension inherent in the life course approach, with links between exposures and outcomes that span many years, or even generations, and (b) an ecological perspective, according to which the contexts in which individuals live are critical, as are changes in those contexts over time.

**Conclusions:**

By explicitly examining longer-term, intergenerational and ecological perspectives, life course concepts can inform and strengthen traditional approaches to injury prevention and control that have a strong focus on proximal factors. The model proposed also serves as a tool to identify intervention strategies that have co-benefits for other areas of health.

## Background

Our understanding of injury, its causes and opportunities for prevention are informed by paradigms drawn from a range of disciplines, including epidemiology, biomechanics, ergonomics and the behavioral and social sciences [1,2]. As many injuries follow acute events and are considered to be relatively sudden in onset [3], it is tempting to focus on the short-term and proximal influences on injury (e.g., speeding as a risk factor for a motor vehicle crash, or the time elapsed to availability of definitive trauma care as a risk factor for injury-related deaths). While such factors are undeniably important, risks of injury are also influenced by living conditions, such as the urban environment, and access to money and other resources [4]. There are a host of distal factors that influence these domains, not all of which are inherently obvious or actively considered as amenable to intervention in many injury prevention strategies.

The life course approach is notable for its longer-term temporal perspective, and is an increasingly influential framework in a range of areas of health, especially preventative medicine [5-7]. In this paper, we examine the models and concepts that have most strongly influenced the field of injury, drawing on seminal articles, book chapters and other prominent resources, and describe the extent to which life course concepts have been addressed in existing approaches to injury. We then summarize the development of and main concepts inherent in the life course approach, and discuss how this approach can be applied to the field of injury. Drawing on this exploration, we offer a model that integrates the two perspectives to address the burden of injury across the life course.

## Common injury concepts

Although a range of different conceptual approaches to injury have been proposed [8], a relatively small number of models and concepts remain central in most injury textbooks [1,9-16] and in current leading teaching resources, such as the TEACH-VIP resource developed by the World Health Organization [17]. We summarize these common concepts here.

William Haddon's seminal contributions to conceptualizing causes and measures to prevent injuries included the matrix that combined the epidemiological triad of host, agent and environment with a temporal dimension covering pre-event, event and post-event phases [18]. In doing so, he drew on John Gordon's earlier work, which described how the causative factors in injury can be categorized according to the three components of the epidemiological triad, and which also described the environment as having physical, biological and social dimensions [10]. An example of how the Haddon matrix [18] can be used to identify potential injury determinants and interventions is shown in Table 1.

**Table 1:** Use of the Haddon matrix to consider targets for interventions to reduce the burden of car crash injuries

	Host/person	Agent/vehicle	Environment (physical and social)
Pre-event	Driver skills and experience Speeding	Roadworthiness of vehicles Visibility of vehicles Good brakes	Graduated driver licensing systems Traffic speed limits
Event	Seatbelt use	Presence of seatbelts, airbags and other vehicle safety features	Median barriers Laws mandating seatbelt use
Post-event	First aid knowledge Presence of co-morbidities	Features that make access easier for emergency services Features that avoid post-crash explosions	Emergency services Treatment and rehabilitation services

Complementing this matrix, Haddon proposed ten types of injury countermeasures which comprise a temporally ordered sequence of approaches designed to control, modify and interrupt the process of energy transfer from the hazard causing injury to the individual(s) that can be affected [19]. Within the range of potential interventions, Haddon advocated that preference should be given to "passive" injury prevention strategies - those that protect the individual without action on the part of that individual - over "active" measures requiring individual action [18].

Runyan introduced a third dimension to the Haddon matrix to aid the decision-making process, introducing criteria such as effectiveness, cost, equity and feasibility [2]. Her approach specifically integrated injury concepts into Bronfenbrenner's social-ecologic model, demonstrating opportunities to intervene across multiple levels of the social environment [20]. Rivara provided a framework describing the process by which basic etiologic research can be translated into improved injury outcomes via the development, testing and implementation of injury interventions [21].

Regardless of the model, the strategies used to prevent injuries are often discussed in terms of the "three Es": *education *(e.g., instructing parents to keep medicines out of children's reach); *engineering *and design of specific agents, products and the physical environment (e.g., designing child-resistant medicine packaging); and *enforcement *(e.g., laws mandating use of child-resistant packaging for hazardous medicines) [1]. The *environment *(physical and social) is sometimes identified as a distinct category, as in the Haddon matrix.

Theories of behavior change have been applied to a number of injury prevention strategies, typically interventions targeting the "host", rather than the "vehicle" or "environment" [22,23]. While not their primary focus, several of these theories explicitly acknowledge the role of environmental barriers and facilitators in behavior change [22]. As apparent from the above, with increasing awareness and emphasis on the environmental context, current concepts in injury prevention promote consideration of "ecological" approaches, in which all parts of a model "influence each other as part of a connected system" [24, p. 557]. The application of such thinking is increasingly evident in fields such as violence prevention [25] and community safety promotion [26,27].

The interactions between people and broader notions of ecology and environment are also fundamental aspects of the life course approach [28,29]. We review the evolution of the latter as a prelude to considering its integration and application to the field of injury.

## The life course approach

Life course epidemiology has been defined as "the study of long term effects on later health or disease risk of physical or social exposures during gestation, childhood, adolescence, young adulthood and later adult life" [30, p. 778]. In addition to examining biological, behavioral and psychosocial pathways to disease that operate across the life course of an individual, the approach invites a consideration of how risks and attributes are transmitted across generations [30]. These concepts are not new or specific to epidemiology, and have been promoted for decades in other disciplines such as psychology, sociology, demography, anthropology and biology [30]. Evidence of correlations between early life factors and adult health was reported over 70 years ago [31], and the link between early childhood factors and adult health was a matter of concern at least as far back as the first years of the 20^th ^century [32,33].

A life course approach in epidemiology was developed largely as a response to the "fetal origins of adult disease" hypothesis in which size at birth (usually measured by birth weight) shows a consistent graded association with cardiovascular risk factors and disease outcomes later in life [34]. When this hypothesis was initially proposed, it was suggested that this association was mediated through some form of biological programming that occurs in early life, particularly during intrauterine development. The concept (referred to more recently as the "developmental origins of health and disease" [35,36]) drew attention to a body of research supporting the notion that several chronic diseases that become apparent in adulthood are linked to patterns of early life growth (both prenatal and postnatal).

This initially challenged the predominant paradigm of chronic disease causation, which tended to focus on proximal determinants and risk factors in adults. The presence of atherosclerotic changes early in life revealed that focusing on risk factors in adulthood (such as smoking and blood pressure), while important, would offer only a partial solution to preventing cardiovascular disease [6].

A life course approach to health and disease largely developed to integrate these two polarized views of disease causation - the focus on distal perinatal risk factors, and the focus on proximal risk factors in adults - by applying a more sophisticated perspective that acknowledges that relevant exposures occur throughout the entire life span. Also fundamental to the modern life course approach is a recognition of the importance of the environment and ecological context [5]. Nancy Krieger's eco-social model shares some of the key principles involved by explicitly acknowledging the importance of the conditions in which people are born, live, work and retire [37]. In other words, "changing individuals need to be studied in a changing world" [[30], p. 781].

Intergenerational influences are also considered actively in a life course approach. For example, research in this domain has identified that child birth size is predicted not only by the characteristics of the mother (e.g. mother's birth size, adult height and parity) but also by characteristics of the grandmother (e.g. grandmother's adult height and parity) [38].

While researchers have previously considered the implications of the life course approach for topics such as suicide [39], child behavior [40], child abuse [41] and combat [42], this approach is not explicitly incorporated in current injury prevention frameworks.

Two main concepts are represented in the modern life course approach: an extended temporal dimension, with links between exposures and outcomes that span many years, or even generations; and an ecological perspective, according to which the contexts in which individuals live are critical, as are changes in those contexts over time. Although some injury control frameworks have incorporated ecological considerations, the extended temporal dimension has not been represented. In the next section, we analyze the relevance of these two main concepts to injury, providing some specific examples that illustrate the concepts.

## Applicability of life course concepts to injury prevention

### Extending concepts regarding "determinants of injury"

Compared to existing injury models, a life course approach explicitly extends the temporal dimension of relevance when considering determinants of injury. While the "pre-event" phase in the Haddon matrix is typically (although not exclusively) applied to a time period that is relatively proximal to the time of the injury event, a life course approach views the relevant determinants as having effects that accrue over a lifetime, with potentially important effects across generations. Intergenerational effects have been demonstrated in the agricultural setting, where parental injury has been associated with a higher risk of subsequent injury to children [43]. This could be related to intergenerational associations in injury risk behaviors, or to the sharing of hazardous environments across generations. There is a need for research that more clearly identifies the mechanisms that mediate or counter the transmission of these risks in different settings.

Similarly, while socioeconomic status is established as an important determinant of injury rates [44], the relevant periods of influence can occur across the life course. As demonstrated for chronic diseases, socioeconomic trajectories across the life course and intergenerational correlations in factors such as income, social class and employment status can mediate important social inequities in health outcomes [4,45,46]. Furthermore, these inequalities must be considered in the context of multiple levels of influence, including the individual and the neighborhood levels [37]. A life course approach highlights the need to address the social inequalities in injury as well as overall injury rates. While the implications at national and regional levels are obvious as attested to by many studies and reviews investigating socioeconomic inequalities in injury, the issues involved are particularly salient at the global level where hazardous environments and inequities in access to resources of many kinds place people living in low- and middle-income countries at much greater risk of injury compared with those in higher-income countries [47]. For example, while childhood drowning in swimming pools have drawn much-needed policy attention in many high-income countries, rates of drowning are not only higher by several orders of magnitude in countries such as Bangladesh and China, but also demand attention to much more challenging contextual issues, such as the risks from flooding of homes and play areas [47].

There are many examples revealing intergenerational correlations in injury risk. Being physically punished as a child is not only associated with being the victim of spousal abuse later in life (an effect across the life course of an individual), it is also associated with abusing, as an adult, one's own child [48]. This abuse, in turn, has lifelong health implications for the victim [41]. The alcohol consumption of parents is correlated with their adolescent children's alcohol consumption [49], and early onset of alcohol drinking (for example, aged under 14 years) not only predicts problem drinking later in life, but also predicts early onset of alcohol drinking in that person's children [50]. Furthermore, while the utility of labeling individuals as "accident-prone" is debatable [51], there is little doubt that the characteristics of the social and physical environments that people live in directly influence their risk of injury. A life course perspective emphasizes the need to see "injury proneness" as representing not fixed, intrinsic personal characteristics, but rather, an opportunity to create safer environments for populations identified as being at higher long-term risk of injury.

### The "added value" for injury interventions

Building on the features noted above, injury prevention strategies with effects that can reach across the life course can be particularly powerful. For example, one home visiting intervention was found not only to reduce child abuse and neglect [52], but also to reduce injury risk factors for children in the intervention group when they were older, in the form of problem drinking in adolescence [53]. Several other social and health outcomes were also improved [54]. This suggests a need to be mindful both of the potential duration of intervention effects, and also the multiple outcomes that may stem from a single intervention. While educational interventions often target proximal injury risk factors, education is also a component of the more distal concept of socioeconomic status, suggesting that some interventions may result in both proximal and distal effects. Home visiting interventions, which can address multiple injury risk factors for several different people in a family, also reflect the importance of employing an ecological perspective that recognizes that people "influence each other as part of a connected system" [24, p. 557]. However, more studies are needed to demonstrate the long-term effects of injury prevention interventions [55].

In the chronic disease sphere, the discovery of atherosclerotic changes as early as the second decade of life led to calls for the "lifelong prevention of atherosclerosis" [6, p. 1129]. Taking a lifelong approach to injury prevention can be considered in the same light. For example, while mortality from falls occurs overwhelmingly in the elderly [56], risk factors for falls accumulate throughout the life course. Trajectories in physical activity start early in life, and physically active children are more likely to become physically active adults [57]. Physical activity has also been shown to be an important component of falls prevention in the elderly [58]. With the neighborhood environment being a recognized determinant of physical activity at all ages [59], exposure to activity-friendly environments early in life could thus be an important environmental determinant of falls in later life. In some situations, the hazardous exposure may interact with other age-related factors to increase the risk of injury over the life course. For example, exposure to high levels of occupational noise affects the cumulative risk of significant hearing impairment, which may manifest later in life due to the additive effects of age-related hearing loss [60]. Community-level factors that can influence drinking initiation among youth [61] can also increase the risk of subsequent alcohol-related unintentional injury in adulthood [62]. These findings indicate the significant benefits that could result from comprehensive injury prevention strategies that consider intervention opportunities both early in life and in later years, with particular awareness of the physical and social environments.

### The ecology of injury

The ecological dimension of the life course approach is an important feature of the proposed model that is, to some extent, distinct from the traditional injury prevention frameworks that consider "host", "vehicle" and "environment", or the "three Es" as specific concepts. Ecological models present the world as a set of interlinked ecosystems whose parts reciprocally influence one another, so both "host" and "vehicle" are constituent parts of these ecosystems. This is exemplified by the dynamic interaction between people, vehicles and the physical environment in a busy road. The "safety in numbers" concept, according to which injury risk for individual cyclists reduces as cyclist numbers increase [63], further illustrates the interdependence within the ecology of the road.

Broader ecological effects also occur through the contribution of transport emissions to climate change, with consequent increases in injury risk factors related to increasingly frequent extreme weather events and other natural disasters [64]. Conversely, the public health response to climate change offers substantial opportunities for injury prevention [65].

Injury interventions should take account of, and capitalize on, these interactions. For example, low-speed zones and traffic calming interventions that enhance the safety of vulnerable road users [63,66] could, in theory, catalyze and mutually reinforce a "safety in numbers" effect for pedestrians and cyclists. Over time, if more people walk and cycle, the safety in numbers effect could increase, further encouraging pedestrians and cyclists, while also galvanizing environmental policy actions that promote and support active modes of travel.

A key feature of interventions such as traffic calming that improve the safety of the road environment for pedestrians and cyclists is their potential to improve opportunities for more active lifestyles that do not inherently increase the risk of serious injuries. Integrating injury prevention with physical activity promotion goals, instead of pursuing each in isolation, can help to manage pre-existing conflicts between these domains of health. For example, concerns about the safety of child pedestrians are likely to have contributed to the decline in walking to school in recent years [67]. While being driven to school reduces that child's pedestrian injury risk, it increases the risks from physical inactivity, indicating a need for alternative solutions that improve safety and physical activity simultaneously. An ecological approach addresses these tensions explicitly by designing and implementing interventions to maximize co-benefits across multiple domains of health. For example, it has been argued that reducing global fossil fuel dependence could simultaneously reduce the large global problems of road traffic injury, obesity and climate change, by transitioning from dependence on motor vehicles to increased use of active transport [68]. The evaluation of interventions also needs to address the multiple outcomes that may stem from interventions, as well as the interdependence between different spheres from the home setting to the city level [69].

## A life course model for injury prevention

The preceding section demonstrates that life course and ecological influences on injury are conceptually plausible and supported by empirical evidence. In this section we propose a model that integrates both injury prevention and life course concepts.

**Figure 1:**
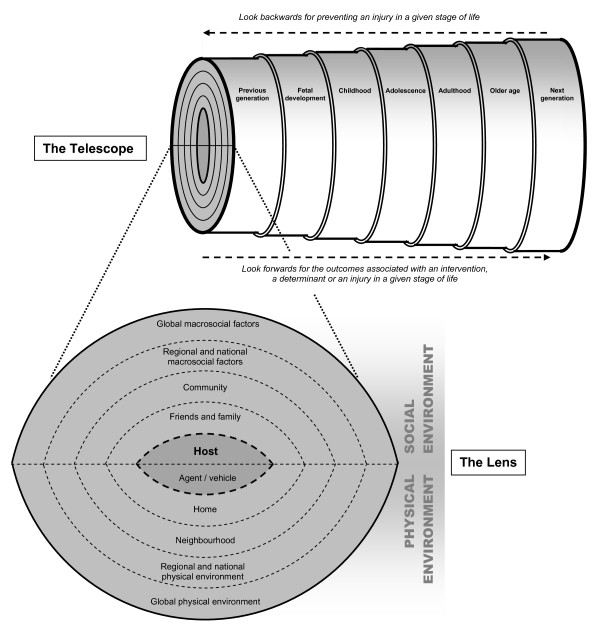
**Ecological model of injury across the life course: the "lens and telescope"**.

As noted earlier, the Haddon matrix remains the most prominent injury conceptual model, with a central place in textbooks and current leading teaching resources. As well as a temporal dimension, the Haddon matrix identifies host, agent and environmental factors (both social and physical) that contribute to injury. The two main concepts represented in the modern life course approach are an extended temporal dimension and an ecological perspective. Figure 1 presents a schematic view of a model that integrates the salient concepts of the Haddon matrix and the life course approach to inform injury prevention.

In comparison with the Haddon matrix (Table 1), this tool emphasizes broader ecological influences and life course and intergenerational determinants of injury. Furthermore, the tool can be used to consider potential long-term effects that can arise both from injuries and from interventions.

Symbolically, the tool takes the form of a telescope and lens - representing its extended time dimension, and its ecological and intergenerational focus. The "lens" contains, at its centre, the host (the person at risk of injury) and the vehicle (through which the transfer of energy occurs, such as a motor vehicle in road traffic injury, or a firearm in the case of gunshot wounds). The host and vehicle are located within and influenced by the social and physical environments, ranging from the home environment to the local community and the global context. This combines the commonly used concepts in the Haddon matrix with the broader levels of environmental influence described by many ecological models [26-28,37,70,71]. These environmental factors are not only of fundamental importance in the causation of injury, but also in the level of functioning and disability after injury, as illustrated by the International Classification of Functioning, Disability and Health [72]. We represent the boundaries between the ecological layers in the tool with dashed lines in recognition of the dynamic interactions between all components of the "lens".

By fitting this "lens" on the end of the "telescope", a life course dimension is added to the tool. As people move through the different stages of the life course, their personal attributes evolve, as do the dynamic social and physical environments with which they interact. All of these factors influence the accumulation of injury risk for individuals, as well as for their families and future generations. Accordingly, each of these factors constitutes a potential target for injury prevention interventions.

An example of the application of the model is provided by the topic of climate change, referred to earlier. The global physical environment (e.g. climate) and macro-social environment (e.g. climate policy) are interdependent. Both influence host factors (e.g. travel behavior), which itself influences road traffic injury risk. Travel behavior also affects greenhouse gas emissions, which contribute to future climate change that will be experienced by the host in later stages of life, as well as by future generations.

## Conclusions

While injuries are typically attributed to discrete events, these are often strongly associated with social and ecological influences including risks accumulated throughout the life course. Some injury risk factors, such as those relating to alcohol use, exposure to violence and socioeconomic status, are also transmissible between generations. Acknowledging the importance of a longer-term perspective on injury - along the lines of a "chronic disease" - we propose a "lens and telescope" model that integrates traditional injury prevention and life course approaches. In doing so, we do not deny the vital significance of addressing the immediate events surrounding an injury, or the final catalysts in pathways that result in injury. Rather, we suggest that explicitly integrating a life course approach helps identify strategies that actively address broader social and ecological determinants as well as achieve co-benefits across multiple health domains for many generations.

## Competing interests

The authors declare that they have no competing interests.

## Authors' contributions

JH and SA drafted the manuscript. All authors contributed to the study conception and analysis, reviewing and revising the article, and approved the final manuscript.

## Pre-publication history

The pre-publication history for this paper can be accessed here:

http://www.biomedcentral.com/1471-2458/11/695/prepub
